# Genome Sequence of *Porphyromonas gingivalis* Strain 381

**DOI:** 10.1128/genomeA.01467-16

**Published:** 2017-01-12

**Authors:** Ryan P. Chastain-Gross, Gary Xie, Myriam Bélanger, Dibyendu Kumar, Joan A. Whitlock, Li Liu, Sarah M. Raines, William G. Farmerie, Hajnalka E. Daligault, Cliff S. Han, Thomas S. Brettin, Ann Progulske-Fox

**Affiliations:** a Department of Oral Biology, University of Florida, Gainesville, Florida, USA; b Center for Molecular Microbiology, University of Florida, Gainesville, Florida, USA; c Interdisciplinary Center for Biotechnology Research, University of Florida, Gainesville, Florida, USA; d Bioenergy and Biome Sciences (B-11), Bioscience Division, Los Alamos National Laboratory, Los Alamos, New Mexico, USA

## Abstract

*Porphyromonas gingivalis* is associated with both oral and systemic diseases. Strain-specific *P. gingivalis* invasion phenotypes do not reliably predict disease presentation during *in vivo* studies. Here, we present the genome sequence of 381, a common laboratory strain, with a single contig of 2,378,872 bp and a G+C content of 48.36%.

## GENOME ANNOUNCEMENT

*Porphyromonas gingivalis* is an anaerobic bacterium (1) that is linked with periodontal disease (2–4) and multiple systemic diseases (5–7). In previous studies (8–11), *P. gingivalis* strains have shown a variety of pathogenic phenotypes *in vitro* and *in vivo*, but underlying genetic mechanisms are poorly defined. Presently, genomic sequences of well-known *P. gingivalis* laboratory strains W83, ATCC 33277, A7436, AJW4, and HG66 have been published (12–16), and W50 is available in the GenBank database (https://www.ncbi.nlm.nih.gov/assembly/483728). Isolated by Anne Tanner at the Forsyth Institute in Boston, Massachusetts, USA (17), 381 is a nonencapsulated strain of *P. gingivalis*. Notably, strains 381 and ATCC 33277 share similar capsule and fimbriae characteristics (18, 19), and both exhibit high invasion efficiencies during *in vitro* infection of human coronary artery endothelial cells (HCAECs) (8, 11). However, 381 induces autophagy and accesses alternative intracellular trafficking pathways, enabling persistence within HCAECs, while ATCC 33277 enters a lysosomal pathway and does not persist (8). In contrast, both strains induce similar inflammatory responses in a mouse abscess infection model (9, 20). This study was undertaken to determine the complete genome sequence of 381 and enable greater understanding of variations in host intracellular trafficking and host inflammatory responses among *P. gingivalis* strains.

*P. gingivalis* strain 381 was obtained from F. Macrina (Virginia Commonwealth University) and grown as previously described (21). Genomic DNA was obtained using the Wizard gDNA purification kit (Promega) and processed to generate shotgun and 3-kb paired-end libraries, which were sequenced using the 454 Life Sciences GS-20 instrument (22) (Roche). 806,578 reads of 123,742,668 bp, with an average read length of 153 bp, were generated.

GS-20 reads were assembled using Velvet version 0.7.63 (https://www.ebi.ac.uk/~zerbino/velvet) (23) and Newbler version 2.3 (Roche) (22). Gaps between contigs were closed by editing in Consed (http://www.phrap.org/consed/consed.html) (24–26) and by PCR-augmented Sanger sequencing. The genome was annotated using the RAST (http://metagenomics.anl.gov) (27) and IMG-ER servers (http://img.jgi.doe.gov/er) (28) and then amended using Gene Prediction Improvement Pipeline software (29).

The genome of *P. gingivalis* 381 has approximately 49-fold coverage and contains a single contig of 2,378,872 bp (G+C content of 48.36%). A total of 2,054 genes were annotated, which included 1,986 predicted coding sequences (CDSs), 53 tRNAs, 12 rRNAs, and one tmRNA. There are 231 subsystems in the genome. Subsystem features observed included: protein metabolism (197), cofactors, vitamins, prosthetic groups and pigments (157), RNA metabolism (64), DNA metabolism (91), carbohydrates (96) and  membrane transport (17).

The annotated *P. gingivalis* 381 genome was compared to *P. gingivalis* strains W83, ATCC 33277, and TDC60 using RAST (27) and IMG-ER (28). All-to-all BLASTp comparisons of predicted protein sequences showed that 381 possesses 64 strain-specific CDSs, all annotated as hypothetical proteins. Of note, 381 is a close relative of ATCC 33277 based on genome clustering analysis, and the gene order is nearly identical between 381 and ATCC 33277, except three minor differences due to inversion, duplication, or deletion of transposable elements.

The availability of the 381 genome enables exploration of how genomic differences among *P. gingivalis* strains offer widely different *in vitro* phenotypes, but may not confer competitive advantage in an animal model of infection.

### Accession number(s).

This genome sequencing project was deposited in GenBank under accession number CP012889. The version described is the first version.
